# Update and, internal and temporal-validation of the FRANCE-2 and ACC-TAVI early-mortality prediction models for Transcatheter Aortic Valve Implantation (TAVI) using data from the Netherlands heart registration (NHR)

**DOI:** 10.1016/j.ijcha.2021.100716

**Published:** 2021-01-23

**Authors:** Hatem Al-Farra, Bas A.J.M. de Mol, Anita C.J. Ravelli, W.J.P.P. ter Burg, Saskia Houterman, José P.S. Henriques, Ameen Abu-Hanna, M.M. Vis, J. Vos, L. Timmers, W.A.L. Tonino, C.E. Schotborgh, V. Roolvink, F. Porta, M.G. Stoel, S. Kats, G. Amoroso, H.W. van der Werf, P.R. Stella, P. de Jaegere, on behalf of the NHR THI Registration Committee

**Affiliations:** a Department of Medical Informatics, Amsterdam UMC, University of Amsterdam, Amsterdam, the Netherlands; b Heart Center, Amsterdam UMC, University of Amsterdam, Amsterdam, the Netherlands; c Netherlands Heart Registration, Utrecht, the Netherlands; d Amphia Hospital, the Netherlands; e St. Antonius Hospital, the Netherlands; f Catharina Hospital, the Netherlands; g Haga Hospital, the Netherlands; h Isala, the Netherlands; i Leeuwarden Medical Center, the Netherlands; j Medisch Spectrum Twente, the Netherlands; k Maastricht University Medical Center, the Netherlands; l Onze Lieve Vrouwe Gasthuis, the Netherlands; m University Medical Center Groningen, the Netherlands; n University Medical Center Utrecht, the Netherlands; o Erasmus University Medical Center, the Netherlands

**Keywords:** Transcatheter Aortic Valve Implantation (TAVI), Prediction models, Model updating, External Validation, Model recalibration, Closed-testing procedure, NHR, Netherlands Heart Registration (“Nederlandse Hart Registratie in Dutch”), Amsterdam UMC, Amsterdam University Medical Center - location AMC (Academic Medical Center), MPM, Mortality Prediction Models, TAVI (TAVR), Transcatheter Aortic Valve Implantation (Replacement), SAVR, Surgical Aortic Valve Replacement, EuroSCORE, European System for Cardiac Operative Risk Evaluation, FRANCE-2, French Aortic National CoreValve and Edwards [15], ACC-TAVI (ACC TVT), American College of Cardiology Transcatheter Valve Therapy, AU-ROC, Area Under the Receiver Operating-Characteristic Curve, AU-PRC, Area Under the Precision-Recall Curve, BSS, Brier-skill score, LVEF, Left Ventricular Ejection Fraction, NYHA, New York Heart Association

## Abstract

**Background:**

The predictive performance of the models FRANCE-2 and ACC-TAVI for early-mortality after Transcatheter Aortic Valve Implantation (TAVI) can decline over time and can be enhanced by updating them on new populations. We aim to update and internally and temporally validate these models using a recent TAVI-cohort from the Netherlands Heart Registration (NHR).

**Methods:**

We used data of TAVI-patients treated in 2013–2017. For each original-model, the best update-method (model-intercept, model-recalibration, or model-revision) was selected by a closed-testing procedure. We internally validated both updated models with 1000 bootstrap samples. We also updated the models on the 2013–2016 dataset and temporally validated them on the 2017-dataset. Performance measures were the Area-Under ROC-curve (AU-ROC), Brier-score, and calibration graphs.

**Results:**

We included 6177 TAVI-patients, with 4.5% observed early-mortality. The selected update-method for FRANCE-2 was model-intercept-update. Internal validation showed an AU-ROC of 0.63 (95%CI 0.62–0.66) and Brier-score of 0.04 (0.04–0.05). Calibration graphs show that it overestimates early-mortality. In temporal-validation, the AU-ROC was 0.61 (0.53–0.67).

The selected update-method for ACC-TAVI was model-revision. In internal-validation, the AU-ROC was 0.63 (0.63–0.66) and Brier-score was 0.04 (0.04–0.05). The updated ACC-TAVI calibrates well up to a probability of 20%, and subsequently underestimates early-mortality. In temporal-validation the AU-ROC was 0.65 (0.58–0.72).

**Conclusion:**

Internal-validation of the updated models FRANCE-2 and ACC-TAVI with data from the NHR demonstrated improved performance, which was better than in external-validation studies and comparable to the original studies. In temporal-validation, ACC-TAVI outperformed FRANCE-2 because it suffered less from changes over time.

## 1 Introduction

Since 2002, the Transcatheter Aortic Valve Implantation (TAVI) was introduced as a less invasive treatment for patients with severe aortic stenosis at high-mortality risk and not candidate for surgical aortic valve replacement (SAVR) [1], [2]. Over the last years, TAVI emerged as a safe and efficacious alternative treatment also for intermediate and low-risk patients with severe aortic stenosis [3], [4].

Proper risk estimation of post-operative early (30-day) mortality following TAVI using mortality prediction models (MPM) may help heart teams in getting insight into the outcome of TAVI procedures and may help to improve the quality of care. In the past, the classical cardiac surgery MPMs, such as the European System for Cardiac Operative Risk Evaluation (EuroSCORE-I and EuroSCORE-II) [5], [6], [7], and the Society of Thoracic Surgeons Predicted Risk of Mortality (STS-PRoM) [8], were used to predict early-mortality after TAVI. However, those classical MPMs had significant limitations in early-mortality prediction after TAVI [9]. Therefore, several TAVI-specific MPMs (such as FRANCE-2 and ACC-TAVI) have been developed for preoperative risk estimation [10], [11]. These TAVI-specific MPMs were externally validated on different TAVI-populations [12], [13], [14], [15]. The models FRANCE-2 and ACC-TAVI have been shown to outperform other validated MPMs on their discrimination performance. However, in those external-validation studies, the predictive performance of both MPMs (FRANCE-2 and ACC-TAVI) was still poor. The discrimination in terms of the Area Under the Receiver-Operating-Characteristics (AU-ROC) was 0.63 and 0.64, respectively; calibration was poor; and accuracy was limited [12], [13].

Besides, MPMs in general may also lose their predictive performance over time due to performance drift [16], [17], [18]. Poor predictive performance could be due to deficiencies in the development methods of the original-models, changes in the population’s characteristics over time (e.g. expanding TAVI indication to low-risk patients), or due to improvements in the intervention procedure. For these reasons, using such MPMs without their adaptation on an external population is suboptimal [18], [19], [20], [21], [22]. Although developing a new MPM from scratch on new datasets is a common practice, especially when the performance of pre-existing models is poor, updating these models can capitalize on information in the pre-existing models [16], [23], [24], [25], [26]. Updating existing prediction models can indeed improve their performance in new populations as demonstrated in various studies [24], [25], [27], [28] and enables reusing the MPMs for their original purposes [13], [29], [30]. Generally, there are three common updating-methods for logistic regression models: updating the intercept, updating the intercept and slope (model-recalibration), or updating all estimated coefficients (model-revision). The closed-testing procedure described by Vergouwe et al. [23] selects the best updating-method.

In spite of the fact that different existing and recently developed TAVI-specific models are available, only a few models were externally validated. For this study, we selected the models FRANCE-2 and ACC-TAVI because they have been externally validated in three external-validation studies and have shown the best performance [12], [13], [14]. One of these three external-validation studies was recently performed on our own NHR population [14]. We hence sought to update the best two externally performing models on our population, and for simplicity did not attempt to update all proposed models.

In this study, we aim to update two TAVI-specific models (FRANCE-2 and ACC-TAVI) for predicting the early-mortality depending on the closed-testing procedure [23]. We perform internal-validation on the updated models using a recent TAVI-cohort from the NHR. To understand the performance of the updated models over time, which best mimics the model’s envisioned usage in clinical practice; we also perform temporal-validation in which the models are tested on a dataset collected prospectively after the models have been updated on earlier data.

## 2 Methods

### 2.1 Study population

In the Netherlands, 16 heart centers perform TAVI procedures. The Dutch heart centers submit patients’ data (including demographics, clinical characteristics, intervention risk factors, procedural details, mortality status, complications, and follow-up data after hospital discharge) to the NHR registry [31]. In total, data from 13 Dutch heart centers, who had the outcome measurement “30-day mortality”, were included in this study. Data from three heart centers were excluded as they did not timely present the outcome measurements. All variables used in each model were obtained from the NHR, including the outcome (early-mortality status), from January 1, 2013, to December 31, 2017. Although the obtained data originate from several centers, in our sample we has no information about which center a patient belongs to due to local privacy regulations.

### 2.2 The prediction models FRANCE-2 and ACC-TAVI

The model FRANCE-2 (French Aortic National CoreValve and Edwards) is an early-mortality risk score. It was developed in 2014, based on the TAVI French registry with 3883 TAVI patients to predict early-mortality after TAVI [10]. As reported on the internal-validation of this model, the AU-ROC of FRANCE-2 was 0.59 (95% CI 0.54–0.64), and both calibration-intercept and calibration-slope did not deviate from their ideal values of zero and one [10]. This MPM was externally validated [13], [14], where the AU-ROC was 0.63 (95% CI 0.60–0.67), the Area Under Precision-Recall Curve (AU-PRC) was 0.09, the Brier-score was 0.044, the Brier-skill score (BSS) was −0.01 and both the calibration-intercept and calibration-slope did significantly deviate from 0 and 1, respectively [13], [14] (see E-component Table 1).

ACC-TAVI was developed in 2016 by the society of thoracic surgeons and the American college of cardiology to predict in-hospital mortality in TAVI patients (n = 20586) in the United States [11]. As reported on the internal-validation of this model, the AU-ROC of ACC-TAVI was 0.66 (95% CI 0.62–0.69), and the calibration-intercept and calibration-slope did not deviate from the ideal values of zero and one, respectively [11]. The stated purpose of this MPM is TAVI patient counselling, quality-of-care improvement, and national monitoring for appropriateness of the selection of patients for TAVI. This MPM was externally validated [13], [14], where the AU-ROC was 0.64 (95% CI 0.61–0.67), the AU-PRC was 0.09, the Brier-score was 0.043, the BSS was 0.002 and only the calibration-intercept did not deviate from zero [14], (E-component Table 1).

### 2.3 Definition of the primary outcome and the used variable predictors

In this study, the primary outcome is the 30-day mortality or early (post-procedural) mortality, which we defined as death within 30-days from the TAVI procedure date.

The variables used in each MPM and the definition of the predictor variables and their corresponding variables in the TAVI-NHR cohort are given in the supplementary material (E-component Tables 2, 3, and 4).

### 2.4 Statistical analysis

Continuous predictor variables are summarized as mean (standard deviation) or median (inter-quartile-range) and were compared using Students’ *t*-test or the Mann-Whitney test as appropriate. Categorical predictor variables are summarized as counts and percentages and were compared using the chi-squared test or Fisher exact test as appropriate. A p-value < 0.05 of a 2-tailed test was considered significant for all analyses. For bootstrapping the 95% confidence interval was calculated using the percentile method.

#### 2.4.1 Missing predictors and missing values

There could be missing predictors and missing values of existing predictors. For missing predictors, and in line with the approach used in other studies [13], [14], [32], if a variable predictor required by an MPM was not registered in the NHR-TAVI cohort, the condition represented by this predictor was assumed to be absent for all patients*.* However, in addition, we performed a sensitivity analysis by simulating the values of the missing predictor variable. In each simulation, we have randomly drawn values, with a probability of 0.5 of each outcome (absent/present), and calculated the predictive performance measures.

If the registered existing variable predictors of FRANCE-2 and ACC-TAVI in the NHR-TAVI cohort have missing values then we assumed that they were missing at random, as we have no specific reason to assume otherwise. Therefore, and in line with the approach in other studies [13], [14], [32], multiple imputations with ten imputed datasets were generated for the missing values using Multiple Imputation by Chained Equations (MICE) [33]. The outcome measure early-mortality was included, as methodologically recommended, in the imputation models of missing variable predictors.

**Fig. 1:**
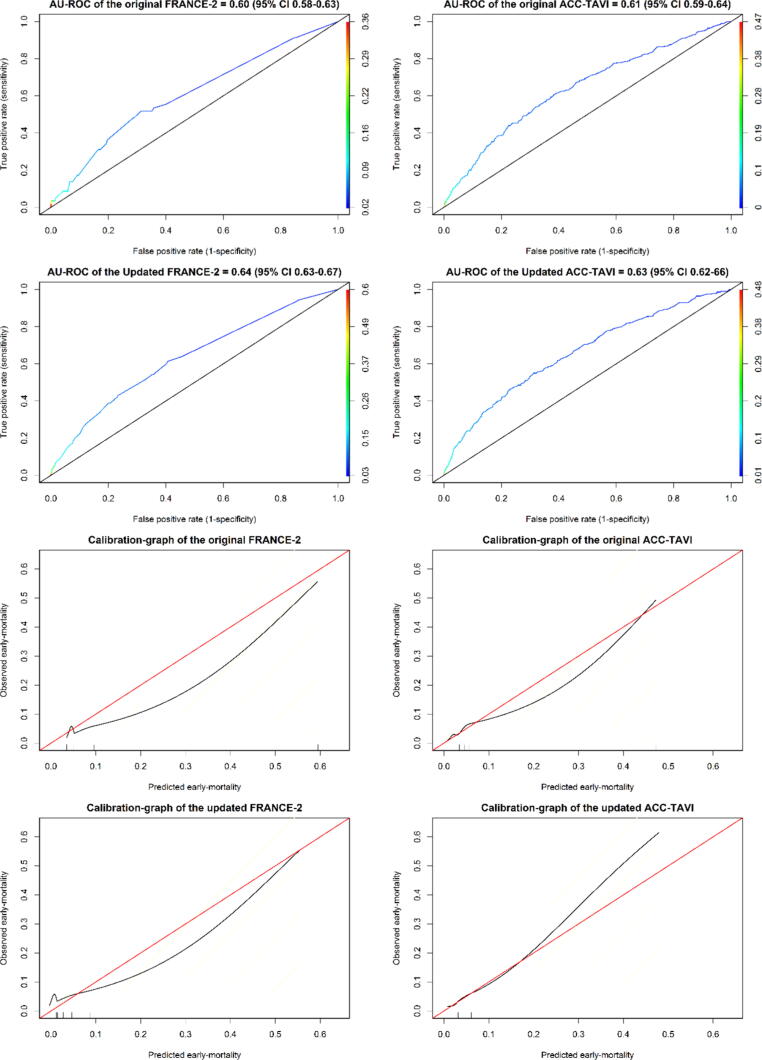
Area under receiver operating characteristic curves and calibration graphs of the original and the updated models France-2 and ACC-TAVI. In the calibration graphs: the vertical axes represent the observed early-mortality, while the horizontal axes represent the predicted probabilities of the early-mortality. Note that there is a high density of cases in the lower range of probabilities.

The flow diagram in E-component Fig. 1 summarizes the following statistical analysis methods.

#### 2.4.2 Selection of the update-method strategy by the closed-testing procedure, and model updating

To select the most appropriate update-method for the two MPMs, we applied the closed-testing procedure of Vergouwe et al. on the whole NHR-TAVI cohort [23]. Application of this procedure will decide on one out of the four following update options: no update; update only the intercept (calibration-in-the-large), update both intercept and slope (logistic calibration); or revise the coefficients of the underlying predictors. Details about these methods appear in E-component Methods 1.

The four update-methods imply an increasing number of estimated parameters. Accordingly, the closed-testing procedure allows the extensiveness of the update to increase progressively from a minimum (no update) to a maximum (model-revision). The procedure involves multiple testing with maintaining approximately the chosen type I error rate by implementing a series of likelihood ratio tests of the updated models against the original-model. The procedure will only select the model-revision method if there is enough evidence that the new regression coefficients are significantly different in the updating population [23].

The update-method that is referred to as model-extension, which considers adding variable predictors other than the original estimated variable predictors, is outside the scope of this paper.

#### 2.4.3 Internal-validation of the update-method strategy

We repeated the multiple imputations and the closed-testing procedure in each of the 1000 bootstrap samples to choose the update-methods in each sample [34]. Specifically, we updated each of the two models (FRANCE-2 and ACC-TAVI) in the bootstrap sample using the corresponding chosen update-method. Consequently, we assessed the optimism corrected performance for our performance measures. The optimism corrected performance was calculated by subtracting the optimism from the apparent model performance, where optimism was based on the difference in the performance of the models trained on the bootstrap samples and tested on the original dataset. We also calculated the proportions of times in which an update method was selected, and the average performance of that chosen method.

#### 2.4.4 Temporal and cross-validation of the updated models

We also validated the predictive performance of the updated MPMs by temporal-validation. Specifically, we updated the models, with the respective selected update-method, on the NHR-TAVI January-2013 up to December-2016 cohort and validated them onward on the cohort from January-2017 up to and including December-2017. This approach reflects the envisioned real-life behavior of the model when facing new patients.

We also performed cross-validation with four folds. This size was chosen so that each fold, and hence the corresponding test set, is about equal to the test set in the temporal-validation approach. Unlike in the temporal-validation approach, in cross-validation, we do not take changes over time (which can denote performance drift) into account. Comparing the results of temporal-validation with cross-validation helps understand whether a model in temporal-validation suffered from changes over time due to drift.

#### 2.4.5 Performance measures

For each of the validation approaches, we used the following performance aspects and their respective measures: discrimination by the Area Under Receiver Operating-Characteristic Curve (AU-ROC); the balance between the positive predictive value (PPV) and the sensitivity by the Area Under Precision-Recall Curve (AU-PRC) [35]; calibration by calibration graphs and the Cox method for inspecting the calibration-intercepts and calibration-slopes [36]; and prediction accuracy by Brier-score and the Brier-skill score (BSS) [37]. For each updated model, we measured the Youden's index (J statistic), which allows to identify the optimal cut-off point of the early-mortality risk probabilities [38] to strike a balance between sensitivity and specificity. Details about these performance measures appear in E-component Methods 2.

All statistical analyses were performed in the R statistical environment version 3.5.1 [39]. Multiple imputations of the dataset were completed using the MICE package. The graphical plots were made using the ggplot2 package. The package pROC was used for constructing the ROC plots and testing the AU-ROCs. The package PRROC was used to construct the PRC plot and obtain the AU-PRCs. The reporting in this study adheres to the TRIPOD checklist for the reporting of multivariable prediction models, the checklist is the E-component Table 8 [40].

## 3 Results

### 3.1 General results

To update the existing FRANCE-2 and ACC-TAVI model, we included 6177 TAVI patients from the NHR-TAVI registration (2013–2017) with an observed early-mortality rate of 4.5% (n = 280) (Table 1). The mean age of the patients was 80.0 years, 51.0% of the patients were female, 7.6% had NYHA class-IV, and 56.0% had NYHA class-III. Urgent TAVI-procedures were 9.0% and emergency procedures were 0.3%. Patients with critical preoperative state had the highest early-mortality risk of 21.1%, followed by patients with NYHA class-IV 9.4%, dialysis with 9.2%, non-transfemoral access route with 8.2%, and urgent procedure-acuity with 7.6% (Table 1). The mean EuroSCORE-II (the estimated early-mortality risk) for the whole population was 5.5%. The mean EuroSCORE-II in the first year (2013) was slightly higher with 5.8%, while in the last year (2017) it was lower with 5.1%. The same pattern has been observed for the mean estimated early-mortality risk when measured by FRANCE-2 (8.2% in 2013, which gradually dropped over the years to 6.9% in 2017), and when measured by ACC-TAVI (4.8% in 2013, which gradually dropped to 4.1% in 2017).

**Table 1:** Patient baseline and procedural characteristics of the study population (n = 6177) stratified according to 30-day postprocedural early mortality.

**Variable**	**Total cases** **N (%)**	**Alive (n = 5897)** **n (%)**	**Early-mortality** **(n = 280)** **n (%)**	**Risk of early-mortality**^**ϕ**^ **(%)**	**P-value**
**Age (year) (mean (SD))**	80.0 (6.90)	79.9 (6.9)	80.9 (28.9)	–	0.036
**Female gender (yes)**	3170 (51.3)	3023 (51.3)	147 (52.5)	4.6	0.731
**BMI kg/m^2^ (mean (SD))**	27.2 (4.88)	27.3 (4.9)	26.4 (9.4)	–	0.006
**eGFR (mean (SD))**	59.1 (21.31)	59.2 (21.7)	55.9 (20.0)	–	0.014
**SPAP (mean (SD))**	31.1 (10.93)	30.9 (10.8)	33.7 (12.0)	–	0.002
**SPAP** ≥ **60 mmHg (yes)**	86	80 (1.3)	6 (2.1)	6.9	
**Chronic lung disease (yes)**	1377 (22.4)	101 (22.2)	76 (27.1)	5.5	0.037
**Critical preoperative state (yes)**	38 (0.6)	30 (0.5)	8 (2.9)	21.1	<0.001
**Dialysis (yes)**	87 (1.5)	79 (1.4)	8 (2.9)	9.2	0.057
**NYHAclass(yes)**					<0.001
NYHA class I	666 (12.5)	646 (12.7)	20 (7.1)	4.5	
NYHA class II	1270 (23.8)	1232 (24.2)	38 (13.6)	2.9	
NYHA class III	2991 (56.1)	2851 (55.9)	140 (50.0)	4.7	
NYHA class IV	405 (7.6)	367 (7.2)	38 (13.6)	9.4	
**Procedure acuity (yes)**					<0.001
Procedure acuity Elective	5415 (90.8)	5200 (91.1)	215 (76.8)	3.9	
Procedure acuity Urgent	536 (9.0)	495 (8.7)	41 (14.6)	7.6	
Procedure acuity Emergency	15 (0.3)	14 (0.2)	1 (0.4)	6.7	
**Unstable angina (yes)**	10 (0.2)	10 (0.2)	0 (0)	0	$
**TAVI access route (yes)**					<0.001
**Transfemoral (TF) access route (yes)**	4926 (79.7)	4744 (80.4)	182 (65)	3.7	
TF Surgical	770 (12.6)	745 (12.8)	25 (8.9)	3.2	
TF Per-cutaneous	2793 (45.8)	2691 (46.2)	102 (36.4)	3.7	
TF Unknown ^φ^	1363 (22.3)	1308 (22.5)	55 (19.6)	4.0	
**Non-transfemoral accessroute(yes)**	1165 (18.8)	1067 (18.1)	96 (34.3)	8.2	
Subclavian access	103 (1.7)	94 (1.6)	9 (3.2)	8.7	
Transapical access	506 (8.3)	462 (7.9)	44 (15.7)	8.7	
Direct aortic access	554 (9.1)	511 (8.8)	43 (15.4)	7.9	
**Acute pulmonaryoedema (yes)**	N.A.	N.A.	N.A.		

Abbreviations: BMI: Body mass index, eGFR: estimated Glomerular Filtration Rate, SPAP: Systolic pulmonary arterial pressure, NYHA: New York Heart Association functional status, TF: Transfemoral.

ϕ: Risk of early-mortality = (the number of death ÷ the total number of cases) x 100. $: Not applicable. φ: TF Unknown: the TAVI access route is also transfemoral access, but the sort (surgical or per-cutaneous) wa was not registered in the dataset. N.A.: Not Available in the NHR-TAVI-cohort.

In the NHR-TAVI cohort, only the predictor variable acute-pulmonary-oedema, which is used in the FRANCE-2, was not registered. The variables systolic pulmonary artery pressure and NYHA class had 35.6% and 13.7% missing values in the TAVI NHR cohort, respectively. The rest of the missing values of predictors were<2%. Nine predictors with missing values were imputed with 10 multiple imputations. E-component Table 5 provides details about the percentage of missing values before imputation.

### 3.2 Performance of FRANCE-2 before and after update

The predicted Mortality of the FRANCE-2 model -as measured before updating the model in our population- was 7.4%. The AU-ROC was 0.60 (95% CI 0.58–0.63). The original model overestimates the early-mortality, as shown in the calibration graph (Fig. 1). The Brier-score was 0.044. The selected update-method after applying the closed-testing was model-intercept-update (Table 2). Performing this update method on the whole dataset resulted in the corresponding final updated model (E-component Table 6a for the final updated intercept of the model).

**Table 2:** Internal-validations of the model update and fitting strategy in each of 1000 bootstrap samples that were applied on the original models FRANCE-2 and ACC-TAVI.

**Performance measures**	**FRANCE-2** **No-update model**	**FRANCE-2** **Model-intercept-update**	**FRANCE-2** **Model-recalibration**	**FRANCE-2** **Model-revision**
**Total number of selected update-methods^$^**	0	339	33	628
**AU-ROC**#**(95% CI)**	0	0.64 (0.63–0.67)	0.67 (0.66–0.68)	0.63 (0.61–0.66)
**Brier-score (95% CI)**	0	0.043 (0.041–0.47)	0.043 (0.041–0.05)	0.043 (0.041–0.05)

**Performance measures**	**ACC-TAVI** **No-update model**	**ACC-TAVI** **Model-intercept-update**	**ACC-TAVI** **Model-recalibration**	**ACC-TAVI** **Model-revision**

**Total number of selected update-methods^$^**	9	0	33	958
**AU-ROC**#**(95% CI)**	0.64 (0.63–0.66)	0	0.65 (0.64–0.68)	0.63 (0.62–0.66)
**Brier-score (95% CI)**	0.042 (0.03–0.05)	0	0.043 (0.041–0.05)	0.043 (0.041–0.05)

Abbreviations: AU-ROC: Area under the Receiver operating characteristic curve, ACC-TAVI: (ACC TVT) American College of Cardiology Transcatheter Valve Therapy, FRANCE-2: French Aortic National CoreValve and Edwards, N.A. Not applicable.

$ Total number of the selected update-methods from the 1000 bootstrap drawn with replacement from the whole NHR-TAVI cohort and having the same size.

# The presented AU-ROC is after adjustment for in-sample optimism.

The predicted mortality of the updated model was 4.8%. The optimism-corrected AU-ROC was 0.64 (0.63–0.67). The updated FRANCE-2 model initially underestimate up to 5% probability then overestimates the early-mortality as shown in the updated FRANCE-2 calibration graph (Fig. 1). Only the calibration-slope did not deviate from its expected value of 1 (Table 2). Brier-score was 0.043 (0.041–0.47).

Repeating the whole model-updating strategy involving the multiple imputations and update-method selection on 1000 bootstrap samples revealed that model-revision was the most selected method (in 62% of the bootstrap samples) (Table 2). This is unlike the model-intercept update method that happened to be selected when deploying the close-test on only the whole cohort. However, the optimism-corrected AU-ROC via bootstrapping of the updated FRANCE-2 (with model-revision) was 0.63 (95% CI 0.61–0.66). Only the calibration-slope did not deviate from its expected value of 1 (Table 2). Details of the results about the Area Under the Precision-Recall-curve, Brier-skill score, and Calibration-in-the-large and calibration-slope appear in E-component Results 1 and 2.

### 3.3 Performance of ACC-TAVI before and after update

The predicted mortality of the original ACC-TAVI model on our population before being updated was 4.4%. The AU-ROC was 0.61 (95% CI 0.59–0.64). The model calibrates well up to predictions of 5%, were most of the predications are. Subsequently, overestimates the early-mortality as shown in the calibration plot (Fig. 1). The Brier-score was 0.043.

Applying the closed-testing method resulted in selecting model-revision as update-method (Table 2). Performing the selected update method on the data results in the corresponding final updated model (E-component Table 6b for the final updated model).

The predicted mortality of the updated model was 4.4%. The optimism-corrected AU-ROC via bootstrapping was 0.63 (95% CI 0.62–0.66). The updated model calibrates well up to predictions of 20%, subsequently, it underestimates the early-mortality proportion as shown in the calibration graph (Fig. 1). Both calibration-intercept and calibration-slope deviated from their ideal values of 0 and 1 (Table 2). The Brier-score was 0.043 (0.041–0.05). Details of the results about the Area Under the Precision-Recall-curve, Brier-skill score, and Calibration-in-the-large and calibration-slope appear in E-component Results 1 and 2.

### 3.4 Results of temporal-validation for both updated models

In the temporal-validation, the training set (years 2013–2016) included data of 4345 patients with an observed early-mortality rate of 5.1%. We updated in this training set each of the original MPMs using the respective update methods that have been selected earlier by applying the closed-testing procedure on the whole cohort. On the validation set (the cohort of the year 2017, n = 1832, observed early-mortality rate = 3.2%).

For the updated model FRANCE-2 (intercept-update), the predicted early-mortality rate in the validation set was 4.6%, the AU-ROC was 0.61 (95% CI 0.53–0.67) (Table 3). For the updated model ACC-TAVI (model revision) the predicted early-mortality rate was 4.6%, and the AU-ROC was 0.65 (95% CI 0.58–0.72) (Table 3). Details of the results about the Area Under the Precision-Recall-curve, Brier-skill score, and Calibration-in-the-large and calibration-slope appear in E-component Results 3.

**Table 3:** Results of temporal-validations and cross-validations ofthe updated-models ACC-TAVI (updated with model-revision) and FRANCE-2 (updated with model-intercept-update). The development sample of the temporal-validation (cohort 2013–2016) n = 4345. The validation sample (cohort 2017) n = 1832. Table is showing the results of the 4-folds cross-validation (n = 1544 per fold).

**Performance measures**	**FRANCE-2** **Model-intercept-update**		**ACC-TAVI** **Model-revision**
**Temporal-validation**			
**AU-ROC**#**(95% CI)**	0.61 (0.53–0.67)		0.65 (0.58–0.72)
**Brier score**	0.031		0.031

**4-folds cross-validation**			

**AU-ROC**#**(95% CI)**	0.63 (0.62–0.67)		0.65 (0.64–0.68)
**Brier-score (95% CI)**	0.043 (0.04–0.5)		0.043 (0.041–0.05)

Abbreviations as in table 2.

# The presented AU-ROC is after adjustment for in-sample optimism.

### 3.5 Results of cross-validation for both updated models

In the 4-folds cross-validation (per fold n = 1544), the AU-ROC updated FRANCE-2 via the intercept-update was 0.63 (95% CI 0.62–0.67). The AU-ROC for the ACC-TAVI (updated with model-revision) was 0.65 (95% CI 0.64–0.68) (Table 3). For both updated models, the calibration-intercept and calibration-slope did not deviate from their ideal values in any of the folds (Table 3).

## 4 Discussion

In this study, we updated and internally and temporally validated the FRANCE-2 and ACC-TAVI prediction models for early-mortality for TAVI patients with contemporary data of TAVI patients from the Netherlands Heart Registration. The update-method for FRANCE-2 was intercept-update and the internally validated AU-ROC was 0.63. The update-method for ACC-TAVI was model-revision and the internally validated AU-ROC was 0.63. After updating the models and on temporal-validation, ACC-TAVI did not have a significantly better AU-ROC than FRANCE-2 (0.65 vs. 0.61, p = 0.06).

Internal-validation of both updated models FRANCE-2 and ACC-TAVI showed AU-ROC (discrimination) to be comparable to the reported performance of their original models [10], [11], and better than the performance reported in the external-validation studies [12], [13], [14]. (Table 2 and E-component Table 1).

Temporal-validation showed improvement in the discrimination ability of the updated models, which was comparable to the original model [11] (Table 3 and E-component Table 1). However, both calibration-intercept and calibration-slope for both updated models have significantly deviated from their ideal values of 0 and 1, respectively.

The updated model FRANCE-2 calibrated poorly in this study. The calibration plot of FRANCE-2 in Fig. 1 shows significant deviations from the ideal calibration in the whole risk range. It underpredicted early-mortality in lower-risk classes (up to 5%) and after that overpredicted early-mortality.

On the other hand, the updated ACC-TAVI calibrated better than the updated FRANCE-2 in this study. The calibration plot of ACC-TAVI in Fig. 1 did not show significant deviations from ideal calibration in the first two deciles of risk (up to 20%). However, the updated ACC-TAVI has underpredicted early-mortality in the higher-risk range, above 20%.

Miscalibration in external-validation studies is common [12], [13], [14], [15]. A common reason could be the improvement of care and/or procedural techniques that took place between the development time of the original MPMs and the time of external-validation. Both FRANCE-2 and ACC-TAVI were developed in the years 2014 and 2016, respectively. Besides, TAVI became a common procedure and the learning curve is likely to have flattened. In addition, the TAVI population’s characteristics have been changing over time (e.g. expanding TAVI indication to intermediate and low-risk patients) instead of the initial predominance of high-risk cases. This is likely the reason behind the reduced mortality risk in this study. A noticeably decrease in early-mortality after TAVI-procedures during that period has been reported [41], [42], [43].

It is worth mentioning that the included patients’ data in this study were collected in a period where most of the candidate patients originated from the high-risk category. However, in 2017 (the last year in our cohort), the European Society of Cardiology guidelines suggested offering TAVI procedures for intermediate-risk patients. This could explain the gradually decreasing mean early-mortality risk, which we measured over the years by the EuroSCORE-II, FRANCE-2 and ACC-TAVI. The inclusion of a relatively lower risk group of patients in the last year increases the heterogeneity of the patient sample in that year. This heterogeneity, in turn, has likely contributed to the improved discriminatory ability of the models in the temporal-validation.

All these factors might affect the predictive performance and calibration of logistic prediction models. Therefore, and unlike in cross-validation, in temporal-validation, both updated models did not perform well when facing population drift. This underscores the importance of implementing a periodic dynamic model update for TAVI-specific MPMs.

Individual updates for the prediction models FRANCE-2 and ACC-TAVI were performed previously by Martin et al. in 2018 [32]. In that study, a “hybrid method” was proposed for updating and aggregating multiple MPMs. A method was used that re-calibrates multiple MPMs using stacked regression while simultaneously revising specific covariates in the final model. They updated both models (FRANCE-2 and ACC-TAVI, in addition to other MPMs) for comparison purposes with their proposed new method. Both updated models (FRANCE-2 and ACC-TAVI) had an AU-ROC of 0.63 and 0.64, respectively [32], which are fairly comparable with our findings.

To the best of our knowledge, our study is new in its use of the closed-testing procedure for selecting appropriate update methods for these two TAVI-specific early-mortality models FRANCE-2 and ACC-TAVI. Apart from the study of Martin et al. [32], we could not find updating studies that report on updating TAVI-specific early-mortality models such as FRANCE-2 or ACC-TAVI. There are, however, model updating studies in cardiology and cardiac surgery for MPMs other than the TAVI-specific MPMs [25], [26], [30], [44]. These studies used either one of the update-methods (intercept update, intercept and slope update, model-revision, or model extension), or apply all of them and choose the model with the best performance without using a formal testing procedure [25], [26], [44]. The utility of deploying the formal closed-testing procedure for selecting an appropriate update method has been motivated by van Calster et al [45]. Of note, in the study [45] and unlike our study, the predictive performance of the updated model was measured instead of repeating the whole update strategy itself as we did in this paper, and as we would recommend.

Our study has several strengths. We used a large multi-center dataset of more than 6000 TAVI patients from a recent national registry dataset. We also used comprehensive predictive performance measures (including the area under the precision-recall curve and Brier-skill Score) to quantify the predictive performance of the updated models. In addition to the internal validation in which the update strategy was repeated in 1000 bootstrap samples, we also performed temporal-validation to inspect the real-life behavior of the updated models when facing new patients; and cross-validation to understand whether this behavior is ascribed to performance drift.

A limitation of this study is that the predictor variable acute-pulmonary-oedema in FRANCE-2 is not registered in the NHR-TAVI registration (E-component Tables 2, 4, and 5). Therefore, in line with other studies [13], [14], [32], we assumed that acute-pulmonary-oedema was absent for all patients. However, to understand whether there is a risk of bias, we performed a sensitivity analysis by simulating the values of the acute-pulmonary-oedema predictor and calculated the performance measures of the updated model. The analysis revealed essentially the same predictive performance measures (E-component Table 7). Another limitation is that the generalisability of the updated models is unknown since we were unable to externally validate the predictive performance in external data. Thus, we recommend researchers to externally validate the models.

We found that ACC-TAVI had the best predictive performance for early-mortality for TAVI patients. For clinical practice, although most of the existing MPMs for TAVI patients are still far from having a good performance, updating the models on new populations does improve their predictive performance, and hence improves their applicability for supporting clinical decision-making.

This study also showed that the updated MPMs suffer from performance drift over time. One should hence, in general, consider a dynamic strategy for updating prediction models, to maintain their relevance to contemporary patient populations. This is a topic that is becoming more pertinent as interventions are increasingly given to lower risk patients [17]. A strategy using statistical process control (SPC) to detect structural deviations from the natural variability in a prediction model’s behavior over time has been suggested as a possible solution to correct for population, and hence, performance drift [46]. In addition, the implications of performance drift on benchmarking have been demonstrated [47], which is useful for quality of care officers.

Different lines of work merit future research. First, one may consider model-extension techniques for updating prediction models, whereby additional predictors (such as anatomical features and dynamic and continuous parameters from ECG, MRI or ECHO) are considered beyond those used in the original model. Second, instead of early-mortality, researchers may consider updating, extending and validating such MPMs with long-term (1-year) mortality as a primary end point. Third, one may consider comparing the updated (and extended) models, with new MPMs developed using different machine learning methods. Fourth, because the estimation of low prevalent outcomes like early-mortality [14], [41], [42], [43] is challenging, one might also look at more prevalent outcomes, such as combining several adverse outcomes (post-operative mortality and complications such as paravalvular leak, major vascular bleeding, stroke and permanent pacemaker implantation) or other patient-relevant outcomes like quality of life. Using additional variables in model extension and update, and by applying machine learning approaches to develop new models, might help identify the best treatment to offer (TAVI vs. SAVR) with the lowest predicted post-operative complication rate [48], assuming that the patient is readily eligible to the given alternatives. Finally, there is a need for more external- and temporal-validation and model updating studies in other countries [30], [49].

## 5 Conclusion

Applying the update-methods and the internal-validation methods on the FRANCE-2 and ACC-TAVI prediction models with data from the NHR-TAVI registration improved the performance of the models to the extent of their original internal validation. Currently, the updated ACC-TAVI with model-revision proved to be the best current tool for early-mortality risk prediction in TAVI patients. However, the predictive performance of the updated models is still suboptimal. The updated models FRANCE-2 and ACC-TAVI are not guaranteed to improve performance in new populations, and hence we recommend that, if possible, other countries and centers consider model updates in their populations as well. Moreover, findings from temporal-validation reinforce the need for implementing a periodic dynamic model update strategy to overcome the effect of performance drift

## Funding sources

This work has no funding sources.

## Author contributions

HF, AAH, AR, and BM contributed to the conception, design, and coordination of the research. The registry commission supervised the collected national TAVI registry data. HF performed all formal analyses with close involvement of AHH and AR. HF wrote the first draft, and AAH and AR contributed to writing the first manuscript. All authors contributed to the critical revision of the article, and the final version was approved by all the authors.

## Declaration of Competing Interest

Hatem Al-Farra, Bas A.J.M. de Mol, Anita C.J. Ravelli, W.J.P.P. ter Burg, Saskia Houterman, José P.S. Henriques, Ameen Abu-Hanna declare no conflict of interest.
