# Supplementary material for: Update and, internal and temporal-validation of the FRANCE-2 and ACC-TAVI early-mortality prediction models for Transcatheter Aortic Valve Implantation (TAVI) using data from the Netherlands heart registration (NHR)
Source: Int J Cardiol Heart Vasc. 2021 Jan 23;32:100716. doi: 10.1016/j.ijcha.2021.100716 (PMC7843396; doi:10.1016/j.ijcha.2021.100716)
Supplement: Supplementary Data 1 [file mmc1.docx]

**E-components Supplementary Methods, Results, Figures and Tables:**

1. **E-component Methods:**

**E-component Methods 1: Details about the update methods**

1) **No update**: in this case, no update is performed and one just uses the original-model as such (1).

2) **Model intercept update** (calibration-in-the-large)**:** In general, this method is used when there is only a difference in the prevalence of the outcome (early-mortality) between the original development and the new population. Updating the model means that a new logistic regression model is created with the following linear predictor *lp updated_model*

*lp _updated_model_ = α_new_ + lp _original_*

where *lp _original_* is the linear predictor of the original model, which is equal to the natural logarithm of the predicted probabilities. We obtained these probabilities using the published regression coefficients of each original MPM. Hence, only the intercept, *α_new_*, is fitted to make the average predicted early-mortality probability equal to the observed overall early-mortality prevalence in the external dataset. In our analysis, we fitted a logistic regression model in each of our 10 imputed datasets with the intercept *α_new_* as the only free parameter and the *lp _original_*_,_ as an offset predictor (i.e. the slope is fixed at unity). For each patient, we averaged the predicted early-mortality obtained from the 10 imputed datasets.

3) **Model intercept and slope update (logistic calibration):** This method is used when the regression coefficients of the original model are over-fitted or under-fitted for the new population. This method will update both the original intercept and the overall calibration-slope. To this end, we fitted logistic regression models in our 10 imputed datasets with *lp original* as the only covariable, as follow:

*lp _updated_model_ = α_new_ + β_overall_ * lp _original_*

4) **Model-revision**: This method updates all the individual regression estimate coefficients of the original model. We used all variable predictors to fit logistic regression models of the following form on the 10 imputed datasets.

*lp _updated_model_ = α _new_ + β _new,1_ * X_1_ + … + β _new,n_ * X_n_*

Where *α _new_* and *β _new,i_* indicate the intercept and the *n* regression coefficients. Subsequently, we pooled the estimated coefficients and standard errors across the 10 models according to Rubin’s rules (2, 3).

**E-component Methods 2: Definitions of the used performance measures:**

**AU-ROC:** The Area Under the Receiver Operating Characteristic Curve is a discrimination measure that gauges the ability of the model to assign higher probability of mortality to those who die than those who live. A value of 0.5 indicates lack of discrimination and a value of 1 indicates perfect discrimination. The AU-ROC is equal in value to the c-index.

**AU-PRC:** The Area Under the Precision-Recall Curve is an aggregate measure of the balance between the positive predictive value (also referred to as precision in the information retrieval community) and sensitivity (also referred to as recall). The higher the value the better the model. The maximum value is 1.

**Brier-score:** This score measures the error in the precision of the predicted probabilities. A perfect model will have a score of 0 and a model that provides the probability of 0.5 to all subjects will have a score of 0.25.

**BSS**: The Brier Skill Score measures the room of improvement in the Brier Score of a model compared to the Brier Score of a non-informative model that provides all

**Calibration-graphs:** these are graphs showing the agreement between the predicted probabilities and the proportion of the events. A well calibrated model will have a graph that is on or close to the diagonal line extending from the point (0, 0) to (1, 1).

**Youden's index: A** statistic equal to sensitivity + specificity -1. The maximum value of the index can be used as a criterion for selecting the optimum cut-off point on the ROC curve. This threshold corresponds to maximizing the distance in the ROC plot from the identity (diagonal) line of the ROC plot. A perfect model would have a Youden's index of 1.

1. **E-component Results:**

**E-component Results 1: Results of Area Under the Precision-Recall-curve, Brier-skill score and Calibration-in-the-large and calibration-slope. Validating the update-method strategy in each of 1000 bootstrap samples that were applied the original models ACC-TAVI and FRANCE-2.**

| **Performance  measures** | **Internal validation of the update-method strategy  in 1000 bootstrap samples on the model FRANCE-2** | | | |
| --- | --- | --- | --- | --- |
|  | **FRANCE-2**  **Original model**  **(No-update model)** | **FRANCE-2**  **Model-intercept-update** | **FRANCE-2**  **Model-recalibration** | **FRANCE-2**  **Model-revision** |
| **Total number of selected  update-methods^$^** | 0 | 339 | 33 | 628 |
| **AU-PRC (95% CI)** | 0 | 0.09 (0.08-0.11) | 0.08 (0.07-0.11) | 0.11 (0.10-0.14) |
| **Brier-skill score (95% CI)** | 0 | 0.005 (0.001-0.01) | 0.004 (0.001-0.02) | 0.015 (0.01-0.03) |
| **Calibration-on-the-large**  **“calibration-intercept”** | N.A. | Not calibrated  -0.41 (-0.69 - -0.13) | Not calibrated  -0.44 (-0.59 - -0.10) | Not calibrated  -0.39 (-0.56 - -0.10) |
| **Logistic-calibration**  **“calibration slope”** | N.A. | Calibrated  1.00 (0.93-1.04) | Calibrated  0.99 (0.93-1.04) | Calibrated  0.99 (0.92-1.03) |
|  |  |  |  |  |
| **Performance  measures** | **Internal validation of the update-method strategy  in 1000 bootstrap samples on the model ACC-TAVI** | | | |
|  | **ACC-TAVI**  **Original model**  **(No-update model)** | **ACC-TAVI**  **Model-intercept-update** | **ACC-TAVI**  **Model-recalibration** | **ACC-TAVI**  **Model-revision** |
| **Total number of selected  update-methods^$^** | 9 | 0 | 33 | 958 |
| **AU-PRC (95% CI)** | 0.09 (0.04-0.11) | 0 | 0.08 (0.04-0.1) | 0.11 (0.1-0.14) |
| **Brier-skill score (95% CI)** | 0.002 (0.001-0.004) | 0 | 0.004 (0.002-0.01) | 0.014 (0.01-0.03) |
| **Calibration-on-the-large**  **“calibration-intercept”** | Not calibrated  -0.39 (-0.61 - -0.12) | N.A. | Not calibrated  -0.38 (-0.62 - -0.09) | Not calibrated  -0.37 (-0.49 - -0.12) |
| **Logistic-calibration**  **“calibration slope”** | Not calibrated  1.16 (1.08-1.32) | N.A. | Not calibrated  1.11 (1.06-1.29) | Not calibrated  1.13 (1.01-1.39) |

Abbreviations: AU-PRC: Area Under the Precision-Recall-curve, ACC-TAVI: (ACC TVT) American College of Cardiology Transcatheter Valve Therapy, FRANCE-2: French Aortic National CoreValve and Edwards, N.A. Not applicable.

$ Total number of the selected methods from the 1000 bootstrap drawn with replacement from the whole NHR-TAVI cohort and having the same size.

# The presented AU-ROC is after adjustment for in-sample optimism

**E-component Results 2: Results of the Youden's index (J statistic) from the temporal validation:**

For the updated-model FRANCE-2 (intercept-update), the best threshold of the early-mortality probabilities was 0.05. At this point, the model’s specificity was 0.69, the accuracy was 0.68, the precision (PPV) was 0.05, and the sensitivity (recall) was 0.52.

For the updated-model ACC-TAVI (model-revision) the best threshold (the optimal cut-off point) of the early-mortality probabilities was 0.04. At this point, the model’s specificity was 0.55, the accuracy was 0.56, the precision (PPV) was 0.05, and the sensitivity (recall) was 0.69.

**E-component Results 3: Results of Area Under the Precision-Recall-curve, Brier-skill score and Calibration-in-the-large and calibration-slope.**

Table is showing the temporal-validation’s results of the updated-models ACC-TAVI (updated with model-revision) and FRANCE-2 (updated with model-intercept-update). The development sample of the temporal-validation (cohort 2013-2016) n= 4345. The validation sample (cohort 2017) n=1832. Table is showing the results of the 4-folds cross-validation (n= 1544 per fold).

| **Performance measures** | **FRANCE-2**  Model-intercept-update | **ACC-TAVI**  Model-revision |
| --- | --- | --- |
| **Temporal validation** | | |
| **AU-PRC (95% CI)** | 0.07 (0.023-0.35) | 0.05 (0.01-0.28) |
| **Brier-skill score** | -0.03 | -0.02 |
| **Calibration-on-the-large**  **“calibration-intercept” (95% CI)** | Not calibrated  -0.43 (-0.68 - -0.15) | Not calibrated  -0.39 (-0.67 - -0.15) |
| **Logistic-calibration**  **“calibration slope” (95% CI)** | Not calibrated  1.12 (1.06-1.23) | Not calibrated  1.13 (1.04-1.22) |
| **4-folds cross validation** | | |
| **AU-PRC (95% CI)** | 0.09 (0.08-0.11) | 0.099 (0.094-0.11) |
| **Brier-skill score (95% CI)** | 0.007 (0.003-0.02) | 0.005 (0.003-0.01) |
| **Calibration-on-the-large**  **“calibration-intercept” (95% CI)**  **Fold 1**  **Fold 2**  **Fold 3**  **Fold 4** | All folds calibrated  -0.19 (-0.45-0.06)  0.10 (-0.14-0.33)  -0.05 (-0.30-019)  0.12 (-0.12-0.35) | All folds calibrated  -0.02 (-0.27-0.22)  -0.02 (-0.27-0.23)  0.07 (-0.18-0.29)  -0.03 (-0.29-0.21) |
| **Logistic-calibration**  **“calibration slope” (95% CI)**  **Fold 1**  **Fold 2**  **Fold 3**  **Fold 4** | All folds calibrated  1.06 (1.15-0.97)  0.97 (1.05-0.89)  1.01 (1.09-0.93)  0.95 (1.03-0.88) | All folds calibrated  0.99 (0.92-1.08)  0.99 (0.91-1.07)  0.99 (0.92-1.08)  1.00 (0.92-1.08) |

Abbreviations: AU-PRC: Area Under the Precision-Recall-curve, ACC-TAVI: (ACC TVT) American College of Cardiology Transcatheter Valve Therapy, FRANCE-2: French Aortic National CoreValve and Edwards,

# The presented AU-ROC is after adjustment for in-sample optimism

1. **E-component Figures:**

**E-component Figure 1:** Flow diagram of the statistical analysis methods

**Model selection (FRANCE-2 and ACC-TAVI)**

*Based on the best predictive performance from
relevant external-validation studies*

**Model updating**

*Use the closed-testing procedure to select the update-method on whole dataset and update (final) model accordingly*

**Validation**

**Internal-validation of the model update
and fitting strategy**

*Repeat the multiple-imputation and selection of update method in 1000 bootstrap samples and correct the final model’s performance based on the estimated optimism*

**Temporal-validation of the updated-models**

*Update models on cohort 2013-2016 (n=4345),
and validate them on cohort 2017 (n=1832)*

**Cross-validation of the updated-models**

*Performed with 4-folds (per fold n=1544) to roughly
match the validation set in the temporal validation*

*Comparison with*

1. **E-component Tables:**

**E-component Table 1:** Comparison of the predictive performance of the models ACC-TAVI and FRANCE-2 in the original studies, in the external validation studies (Martin et. al) [5] and (Al-Farra et. al) [6], and in this study.

| **FRANCE-2** | | | | | |
| --- | --- | --- | --- | --- | --- |
| **Performance measures** | **Original study** (7)  n= 3833 | **External validation studies** | | **Updated FRANCE-2**  Model-intercept-update | |
|  |  | **UK-STUDY** (5) n= 6676 | **NHR-STUDY (6)** n= 6177 | **Internal validation** n= 6177 | **Temporal validation** validation set  (year 2017) n= 1832 |
| **Observed early mortality risk (n)** | 10.0% (382) | 5.4% (360) | 4.5% (280) | 4.5% (280) | 3.2% (58) |
| **Estimated early-mortality risk** | Reported as good concordance from  observed risk | 9.2%  (overestimation) | 7.4%  (overestimation) | - | 4.6% |
| **AU-ROC (95%CI)** | 0.59 (0.54-0.64) | 0.62 (0.59-0.65) | 0.63 (0.60-0.67) | 0.64 (0.63-0.67) | 0.61 (0.53-0.67) |
| **AU-PRC** | N.A. | N.A. | 0.09 | 0.09 (0.08-0.11) | 0.07 (0.023-0.35) |
| **Brier-score** | N.A. | 0.053 | 0.044 | 0.043 (0.041-0.47) | 0.031 |
| **Brier-skill score** | N.A. | N.A. | -0.01 | 0.005 (0.001-0.01) | -0.03 |
| **Calibration-intercept (95%CI)** | Calibrated | −0.60 (−0.71- −0.49) | -0.53 (-0.66- -0.41) | Not calibrated  -0.41 (-0.69 - -0.13) | Not calibrated  -0.43 (-0.68 - -0.15) |
| **Calibration-slope (95%CI)** | Calibrated | 0.69 (0.53-0.86) | 1.21 (1.16-1.26) | Calibrated  1.00 (0.93-1.04) | Not calibrated  1.13 (1.04-1.22) |
|  |  |  |  |  |  |
| **ACC-TAVI** | | | | | |
| **Performance measures** | **Original study (4)**  n= 20586 Validation set n = 6868 | **External validation studies** | | **Updated ACC-TAVI**  Model-revision | |
|  |  | **UK-STUDY** (5) n= 6676 | **NHR-STUDY (6)** n= 6177 | **Internal validation**  n= 6177 | **Temporal validation**  validation set  (year 2017) n= 1832 |
| **Observed early mortality risk (n)** | 4.4% (300) | 5.4% (360) | 4.5% (280) | 4.5% (280) | 3.2% (58) |
| **Estimated early-mortality risk** | Reported with no significant difference from observed risk | 5.2% | 4.4% | 4.6% | 4.6% |
| **AU-ROC (95% CI)** | 0.66 (0.62-0.69) | 0.64 (0.60-0.67) | 0.64 (0.61-0.67) | 0.63 (0.62-0.66) | 0.65 (0.58-0.72) |
| **AU-PRC** | N.A. | N.A. | 0.09 | 0.11 (0.1-0.14) | 0.05 (0.01-0.28) |
| **Brier-score** | N.A. | 0.051 | 0.043 | 0.043 (0.041-0.05) | 0.031 |
| **Brier-skill score** | N.A. | N.A. | 0.002 | 0.014 (0.01-0.03) | -0.02 |
| **Calibration-intercept (95%CI)** | Calibrated | 0.04 (−0.07-0.15) | 0.04 (-0.08-0.16) | Not calibrated  -0.37 (-0.49 - -0.12) | Not calibrated  -0.39 (-0.67 - -0.15) |
| **Calibration-slope (95%CI)** | Calibrated | 0.67 (0.52-0.82) | 0.98 (0.94-1.01) | Not calibrated  1.13 (1.01-1.39) | Not calibrated  1.13 (1.04-1.22) |

Abbreviations: AU-ROC: Area under the Receiver operating characteristic curve, AU-PRC: Area Under the Precision-Recall-curve, ACC-TAVI: (ACC TVT) American College of Cardiology Transcatheter Valve Therapy, FRANCE-2: French Aortic National CoreValve and Edwards, N.A. Not applicable.

**E-component Table 2:** The variables used in the original models ACC-TAVI and FRANCE-2

| **Variable** | **FRANCE-2**  **(10 variables)** | **ACC-TAVI**  **(9 variables)** |
| --- | --- | --- |
| **Age (mean (SD))** | **✓** | **✓** |
| **Gender (female) (yes)** |  |  |
| **sPAP^a^ (mean (SD))** | **✓** |  |
| **sPAP^a^ >60 mm Hg (yes)** | **✓** |  |
| **Chronic lung disease (yes)** | **✓** | **✓** |
| **Critical preoperative state (yes)** | **✓** |  |
| **Dialysis (yes)** | **✓** | **✓** |
| **Functional status NYHA (yes)** |  |  |
| **NYHA class IV** | **✓** | **✓** |
| **Procedure acuity (yes)** |  |  |
| **Procedure acuity Elective 2** |  | **✓** |
| **Procedure acuity Urgent 3** |  | **✓** |
| **Procedure acuity Emergency 4** |  | **✓** |
| **TAVI access route (yes)** |  |  |
| **Non Transfemoral access (yes)** |  | **✓** |
| **Subclavian access** | **✓** |  |
| **Transapical access** | **✓** |  |
| **Direct aortic access** | **✓** |  |
| **Other access** | **✓** |  |
| **BMI (mean (SD))** |  |  |
| **BMI <18 (yes)** | **✓** |  |
| **BMI 18-30 (yes)** | **✓** |  |
| **eGFR (mean (SD))** |  | **✓** |
| **Acute pulmonary oedema** | **✓** |  |

Abbreviations: sPAP: Systolic pulmonary arterial pressure, NYHA: New York Heart Association, BMI: Body mass index, eGFR: estimated Glomerular Filtration Rate, TF: Transfemoral.

**E-component Table 3:** Variable matching between the ACC-TAVI model and the 2013-2017 NHR-TAVI registration

| **ACC-TAVI Variable** | **NHR TAVI** | **Mapped TAVI Values** |
| --- | --- | --- |
| **Age per 5-year increments** | Age at operation | Age divided by 5 rounded down to whole number |
| **eGFR**  Calculated based on age, sex, race, pre-procedure creatinine and requirement of preprocedural dialysis. | Age at operation  Sex,  Creatinine | Calculated by the Modification of Diet in Renal Disease formula |
| **Dialysis vs no dialysis**  The patient is currently undergoing either haemodialysis or peritoneal dialysis on an ongoing basis as a result of renal failure. | On dialysis | Yes |
| **NYHA class IV**  The patient has cardiac disease with dyspnoea at rest that increases with any physical activity, resulting in inability to perform any physical activity without discomfort. | NYHA status | Symptoms at rest or minimal activity |
| **Severe chronic lung disease**  The patient has a history of severe chronic lung disease, defined as FEV1 <50% predicted, and/or room air pO2 < 60 or Room Air pCO2 > 50. | History of pulmonary disease | COAD/emphysema  Asthma  Other significant pulmonary disease |
| Non-femoral access site  The procedure was performed using a femoral access site for the valve sheath. | Delivery approach | **Not**:  “Femoral – percutaneous” or  “Femoral – surgical” |
| **Procedure acuity status category:** |  |  |
| **Acuity status category 2:**  The patient meets both of the following criteria:   1. Procedure status is urgent. 2. No pre-procedure shock, inotropes, mechanical assist device, or cardiac arrest are required. | Critical Pre-Operative Status  Previous MI  Procedure acuity | Yes if  Procedure acuity = “Urgent”  AND  Critical pre-operative status = “No”  AND  Previous MI is No MI |
| **Acuity status category 3:**  The patient meets all three of the following criteria:   1. Procedure status is elective or urgent. 2. Patient had pre-procedure shock, inotropes, or mechanical assist device. 3. No prior cardiac arrest within 24 hours of procedure. | Critical Pre-Operative Status  Previous MI  Procedure acuity | Yes if  Procedure acuity = “Urgent”/ “Elective”  AND  Critical pre-operative status = “Yes”  AND  Previous MI is not recorded as 6-24 hours or <6 hours |
| **Acuity status category 4:**  The patient meets either one or both of the following:   1. Procedure status is emergency or salvage; or 2. Patient had prior cardiac arrest within 24 hours of operation**.** | Critical Pre-Operative Status  Previous MI  Procedure acuity | Yes if  Procedure acuity = “Emergency”/ “Salvage”  OR  Previous MI = MI 6-24 hours or MI <6 hours |

**E-component Table 4:** Variable matching between the FRANCE-2 model and the 2013-2017 NHR-TAVI registration

| **FRANCE-2 Variable** | **NHR-TAVI** | **Mapped TAVI Values** |
| --- | --- | --- |
| Age | Age | Age split into categories as per model |
| BMI | Weight and Height | ((Weight)/(Height^2)) split into categories as per model |
| Respiratory Insufficiency | History of pulmonary disease | COAD/emphysema  Asthma  Other significant pulmonary disease |
| Acute pulmonary oedema^±^ | N/A | N/A |
| NYHA Class IV | NYHA dyspnoea status | Symptoms at rest or minimal activity |
| Dialysis | On Dialysis | Yes |
| Pulmonary hypertension | PA Systolic > 60mmHg | Yes |
| Critical preoperative state | Critical Pre-Operative Status | Yes |
| Transapical Access | Delivery Approach | Transapical |
| Other Access | Delivery Approach | Any option other than Transapical or Transfemoral access |

± Assume not present for all patients

**E-component Table 5:** Predictor variables with missing (%) values, baseline characteristics before imputation in the NHR-TAVI cohort (6177 patients)

| **Variable** | **Missing values in NHR-TAVI cohort** | |
| --- | --- | --- |
|  | **Number of missing** | **%** |
| **estimated Glomerular Filtration Rate (eGFR)** | 30 | 0.5 |
| **Chronic lung disease** | 32 | 0.5 |
| **Access route** | 83 | 1.3 |
| **Critical preoperative state** | 93 | 1.5 |
| **Body mass index (BMI)** | 125 | 2.0 |
| **Dialysis** | 201 | 3.3 |
| **Procedure acuity** | 211 | 3.4 |
| **Functional NYHA class (I, II, III, and IV)** | 845 | 13.7 |
| **Systolic Pulmonary Artery Pressure (mm Hg)** | 2203 | 35.6 |
| **Acute pulmonary oedema** | Not registered in the TAVI-NHR cohort | -- |

**E-component Table 6 a:** The new estimated new updated intercept of the model FRANCE-2

| **Variables predictors of the updated model FRANCE 2** | **Coefficients** |
| --- | --- |
|  |  |
| **New updated intercept from this study,  to be used with the original coefficients** | -3.84 |
| **Original published coefficients [7]** |  |
| **Intercept (old)** | -3.32 |
| **Age** | 0.42 |
| **BMI <18** | 0.82 |
| **BMI 18-30** | 0.41 |
| **NYHA class IV** | 0.58 |
| **Acute pulmonary oedema** | 0.47 |
| **SPAP >60 mm Hg** | 0.37 |
| **Critical preoperative state** | 0.87 |
| **Chronic lung disease** | 0.5 |
| **Dialysis** | 1.06 |
| **Transapical access** | 0.7 |
| **Direct aortic, Subclavian or Other access** | 0.78 |

Abbreviations: BMI: Body mass index, eGFR: estimated Glomerular Filtration Rate, SPAP: Systolic pulmonary arterial pressure, NYHA: New York Heart Association, TF: Transfemoral.

**E-component Table 6 b:** The new estimated coefficients for the final updated model ACC-TAVI

| **Variables predictors of the  updated model ACC-TAVI** | **New**  **coefficients** | **Std. Error** | **Std. Error 95%CI** | | **OR** | **OR 95%CI** | | **P. value** | **Published coefficients [4]** |
| --- | --- | --- | --- | --- | --- | --- | --- | --- | --- |
|  |  |  | **2.5 %** | **97.5 %** |  | **2.5 %** | **97.5 %** |  |  |
| **Intercept** | -5.93 | 0.822 | -7.57 | -4.35 | 0.00 | 0.00 | 0.01 | 5.360E-13 | -4.72976 |
| **Age per 5-y increments** | 0.17 | 0.049 | 0.07 | 0.26 | 1.18 | 1.07 | 1.30 | 0.001 | 0.12185 |
| **eGFR per 5-U increments** | -0.02 | 0.015 | -0.05 | 0.01 | 0.98 | 0.95 | 1.01 | 0.247 | -0.06933 |
| **Dialysis** | 0.54 | 0.393 | -0.29 | 1.26 | 1.72 | 0.75 | 3.54 | 0.168 | 1.17932 |
| **NYHA class IV** | 0.59 | 0.182 | 0.22 | 0.94 | 1.80 | 1.25 | 2.55 | 0.001 | 0.22304 |
| **Chronic lung disease** | 0.29 | 0.140 | 0.01 | 0.56 | 1.34 | 1.01 | 1.75 | 0.037 | 0.51084 |
| **Procedure access site** |  |  |  |  |  |  |  |  |  |
| **Non-femoral access site** | 0.84 | 0.133 | 0.57 | 1.10 | 2.31 | 1.77 | 2.99 | 0.000 | 0.67347 |
| **Procedure Acuity** |  |  |  |  |  |  |  |  |  |
| **Acuity category 2** | 0.61 | 0.185 | 0.23 | 0.96 | 1.84 | 1.26 | 2.61 | 0.001 | 0.4507 |
| **Acuity category 3** | 1.47 | 0.485 | 0.42 | 2.35 | 4.33 | 1.52 | 10.52 | 0.003 | 0.99269 |
| **Acuity category 4** | 0.83 | 0.314 | 0.16 | 1.40 | 2.28 | 1.17 | 4.06 | 0.009 | 1.20737 |

Abbreviations: BMI: Body mass index, eGFR: estimated Glomerular Filtration Rate, SPAP: Systolic pulmonary arterial pressure, NYHA: New York Heart Association, TF: Transfemoral.

**E-component Table 7:** Sensitivity analysis by simulating the values of the absent variable predictor (acute-pulmonary-oedema) and calculated the performance measures of the updated-model in each of 1000 bootstrap samples

| **Performance measures** | **FRANCE-2-NHR**  Model-intercept-update |
| --- | --- |
| **AU-ROC^#^ (95% CI)** | 0.61 (0.52-0.65) |
| **AU-PRC** | 0.06 |
| **Brier score** | 0.031 |
| **Brier-skill score** | -0.04 |
| **Calibration-on-the-large**  **“calibration-intercept” (95% CI)** | Not calibrated  -0.5 (-0.7 - -0.2) |
| **Logistic-calibration**  **“calibration slope” (95% CI)** | Not calibrated  1.13 (1.04-1.22) |

**E-component Table 8: TRIPOD Checklist: Prediction Model Validation**

| **Section/Topic** | **Item** | **Checklist Item** | **Page** |
| --- | --- | --- | --- |
| **Title and abstract** | | | |
| Title | 1 | Identify the study as developing and/or validating a multivariable prediction model, the target population, and the outcome to be predicted. | 1* |
| Abstract | 2 | Provide a summary of objectives, study design, setting, participants, sample size, predictors, outcome, statistical analysis, results, and conclusions. | 2 |
| **Introduction** | | | |
| Background and objectives | 3a | Explain the medical context (including whether diagnostic or prognostic) and **rationale** for developing or validating the multivariable prediction model, including references to existing models. | 3 |
|  | 3b | Specify the objectives, including whether the study describes the development or validation of the model or both. | 3 |
| **Methods** | | | |
| Source of data | 4a | Describe the study design or source of data (e.g., randomized trial, cohort, or registry data), separately for the development and validation data sets, if applicable. | ✓ |
|  | 4b | Specify the key study dates, including start of accrual; end of accrual; and, if applicable, end of follow-up. | ✓ |
| Participants | 5a | Specify key elements of the study setting (e.g., primary care, secondary care, general population) including number and location of centres. | ✓ |
|  | 5b | Describe eligibility criteria for participants. | ✓ |
|  | 5c | Give details of treatments received, if relevant. | NA |
| Outcome | 6a | Clearly define the outcome that is predicted by the prediction model, including how and when assessed. | ✓ |
|  | 6b | Report any actions to blind assessment of the outcome to be predicted. | ✓ |
| Predictors | 7a | Clearly define all predictors used in developing or validating the multivariable prediction model, including how and when they were measured. | ✓ |
|  | 7b | Report any actions to blind assessment of predictors for the outcome and other predictors. | ✓ |
| Sample size | 8 | Explain how the study size was arrived at. | ✓ |
| Missing data | 9 | Describe how missing data were handled (e.g., complete-case analysis, single imputation, multiple imputation) with details of any imputation method. | ✓ |
| Statistical analysis methods | 10c | For validation, describe how the predictions were calculated. | ✓ |
|  | 10d | Specify all measures used to assess model performance and, if relevant, to compare multiple models. | ✓ |
|  | 10e | Describe any model updating (e.g., recalibration) arising from the validation, if done. | ✓ |
| Risk groups | 11 | Provide details on how risk groups were created, if done. | ✓ |
| Development vs. validation | 12 | For validation, identify any differences from the development data in setting, eligibility criteria, outcome, and predictors. | ✓ |
| **Results** | | | |
| Participants | 13a | Describe the flow of participants through the study, including the number of participants with and without the outcome and, if applicable, a summary of the follow-up time. A diagram may be helpful. | ✓ |
|  | 13b | Describe the characteristics of the participants (basic demographics, clinical features, available predictors), including the number of participants with missing data for predictors and outcome. | ✓ |
|  | 13c | For validation, show a comparison with the development data of the distribution of important variables (demographics, predictors and outcome). | ✓ |
| Model performance | 16 | Report performance measures (with CIs) for the prediction model. | ✓ |
| Model-updating | 17 | If done, report the results from any model updating (i.e., model specification, model performance). | ✓ |
| **Discussion** | | | |
| Limitations | 18 | Discuss any limitations of the study (such as nonrepresentative sample, few events per predictor, missing data). | ✓ |
| Interpretation | 19a | For validation, discuss the results with reference to performance in the development data, and any other validation data. | ✓ |
|  | 19b | Give an overall interpretation of the results, considering objectives, limitations, results from similar studies, and other relevant evidence. | ✓ |
| Implications | 20 | Discuss the potential clinical use of the model and implications for future research. | ✓ |
| **Other information** | | | |
| Supplementary information | 21 | Provide information about the availability of supplementary resources, such as study protocol, Web calculator, and data sets. | ✓ |
| Funding | 22 | Give the source of funding and the role of the funders for the present study. | ✓ |

** Please note that the exact related page location could be filled just after the final acceptance*

**References**

1. Steyerberg E. Clinical Prediction Models, A Practical Approach to Development, Validation, and Updating. New York NY: Springer Science & Business Media, LLC; 2009.

2. Rubin DB. Multiple Imputation for Nonresponse in Surveys: Wiley; 2004.

3. Barnard J, Rubin DB. Small-Sample Degrees of Freedom with Multiple Imputation. Biometrika. 1999;86(4):948-55.

4. Edwards FH, Cohen DJ, O'Brien SM, Peterson ED, Mack MJ, Shahian DM, et al. Development and Validation of a Risk Prediction Model for In-Hospital Mortality After Transcatheter Aortic Valve Replacement. JAMA Cardiol. 2016;1(1):46-52.

5. Martin GP, Sperrin M, Ludman PF, de Belder MA, Gale CP, Toff WD, et al. Inadequacy of existing clinical prediction models for predicting mortality after transcatheter aortic valve implantation. Am Heart J. 2017;184:97-105.

6. Al-Farra H, Abu-Hanna A, de Mol B, Ter Burg WJ, Houterman S, Henriques JPS, et al. External validation of existing prediction models of 30-day mortality after Transcatheter Aortic Valve Implantation (TAVI) in the Netherlands Heart Registration. Int J Cardiol. 2020.

7. Iung B, Laouenan C, Himbert D, Eltchaninoff H, Chevreul K, Donzeau-Gouge P, et al. Predictive factors of early mortality after transcatheter aortic valve implantation: individual risk assessment using a simple score. Heart. 2014;100(13):1016-23.
